# The aesthetic experience of critical art: The effects of the context of an art gallery and the way of providing curatorial information

**DOI:** 10.1371/journal.pone.0250924

**Published:** 2021-05-28

**Authors:** Magdalena Szubielska, Kamil Imbir

**Affiliations:** 1 Institute of Psychology, The John Paul II Catholic University of Lublin, Lublin, Poland; 2 Faculty of Psychology, University of Warsaw, Warsaw, Poland; Liverpool Hope University, UNITED KINGDOM

## Abstract

The aim of our research was to investigate the influence of the situational context of presenting contemporary critical artworks (in an art gallery vs in a laboratory setting) and the way in which one is acquainted with contextual information, i.e. a curatorial description (reading it on one’s own vs listening to it vs a lack of curatorial information), on the reception of critical art. All experimental stimuli were exemplars of contemporary art which raise current controversial social and political issues. Non-experts in the field of art were asked to rate their emotional reactions on non-verbal scales and estimate their liking and understanding of the artworks. As predicted, the art gallery context increased both the experience of aesthetic emotions–in terms of valence, arousal, subjective significance, and dominance and aesthetic judgements–in terms of liking. Thus, for critical art (i.e. current artworks which critically address serious, up-to-date issues) the situational context of the gallery increased the aesthetic experience–which is in line with previous studies on the gallery (or museum) effect. Curatorial information increased understanding, so non-experts seem to need interpretative guidance in the reception of critical art. Subjective significance was higher in the reading of curatorial information condition than the listening to curatorial information condition or the control condition (a lack of curatorial information). It seems, therefore, that art non-experts have a better understanding of critical art after being exposed to the curatorial description, but this does not result in an increase in liking and aesthetic emotions. Probably this is because the curatorial description allows one to grasp the difficult, often unpleasant issue addressed by critical art.

## Introduction

Artists are increasingly expressing themselves through their work about the problems of the world in which we live. They criticise the observed social and political situation or climate changes in the world. We believe that an interesting and important research area in experimental aesthetics is to find out what experiences critical art evokes in society among people who are not art experts. Does viewing pieces of critical art outside the gallery make as strong an impression on the recipients as viewing genuine artworks? How does the availability of curatorial information change aesthetic emotions and judgements? Exploring the answers to these questions will allow us to understand under what conditions critical art can become a social change tool.

The current study focuses on the influence of the situational context of the reception of contemporary critical artworks and the manner of delivering the curatorial information about these artworks on emotional reaction and aesthetic judgement. We manipulated variables that have an influence on largely automatic, but also top-down processing in art perception [1]. Leder and colleagues’ model of information-processing of aesthetic appreciation and aesthetic judgements [2] was adopted as the theoretical basis for our study. According to the model, two relatively independent outputs of art processing are aesthetic emotion and aesthetic judgements. At every stage of art processing (from automatic to deliberate), a viewer can continuously access the outcome of affective evaluation (i.e. emotional reaction). Aesthetic judgements are the result of the evaluation of the cognitive mastering stage, i.e. they depend on whether a viewer finds their own interpretation of the artwork as cognitively satisfactory or not. The reception of contemporary art is driven by the need for understanding since most contemporary art is cognitively challenging. Lack of understanding correlates with an unfavourable evaluation of art and may cause the viewer to have negative aesthetic emotions (especially in the case of contemporary art that is not visually pleasing). On the other hand, the meaning-making process, if it ends satisfactorily, is self-rewarding.

### Aesthetic reception of contemporary critical art

Contemporary art often takes a critical approach to current difficult and controversial political topics or social issues (e.g. minority or animal rights, the refugee crisis, climate change). Some recent research has shown how visual activist art, engaged in the problems of the contemporary world, might change the attitudes of viewers and be a driver of social change [3–5].

In Poland (where the data for the current study were collected), critical art has shown very dynamic development. However, the public has mostly reacted negatively to this kind of art and has rejected it, seeing it as devoid of value [6–9]. In the Polish context, the term "critical art" was first used in reference to the practices of the members of the KwieKulik artistic collective (especially those practices occurring during the 1970s and 1980s), who manifested a critical stance towards the reality of Poland under communism [10] (in fact, Poland is the country where critical art, defined in the narrow sense, was born). This term was also used to refer to the art of Polish artists (e.g. Katarzyna Kozyra, Artur Żmijewski) who explored the issue of artistic freedom (mainly by experimenting with the body) in the light of the events of 1989 –after the fall of communism [9]. They were characterised by intransigence, and exposing the habits of a society that is insensitive to suffering, inequality and discrimination. In the broader view (this is how we define critical art in the current study), contemporary critical art may be defined as art critically addressing acute contemporary socio-political issues (e.g. prejudices against refugees or persecution of non-heteronormative people). Very often critical art is conceptual, i.e. art which is intellectually stimulating but visually unrewarding [1]. An example of an exhibition presenting such art is the *Three Plagues* exhibition, the aim of which was to exhibit artistic statements that are critical of readiness for and acquiescence to violence, which is analysed in this study.

This is not only the case in Poland–provocative or challenging artworks evoke negative emotions (like anger or disgust) because of a lack of education in the field of contemporary art, which results in the negation of the artistic value of art currently being created [11–13]. Referring to a model of aesthetic appreciation and aesthetic judgements, we can explain that by a failure to provide a satisfying interpretation of critical artworks, which causes a decrease in aesthetic pleasure [2]. Furthermore, the appraisal model of aesthetic emotions more profoundly explains how a personal evaluation of an artwork causes specific emotions, including negative emotions of anger, disgust, and contempt [11–14]. These so-called hostile emotions may cause rejection and motivate aggression and violence in naive viewers who are faced with confrontational contemporary artworks (from time to time there are reports in the media that, e.g., some blasphemous artwork was attacked or destroyed by an appalled viewer or people took part in turbulent demonstrations against an exhibition that offends their religious values). Why do non-expert viewers with conservative tastes experience such hostile emotions towards contemporary artworks? According to the appraisal theory of aesthetic emotions, feelings towards art are connected with cognitive processes. For instance, viewers experience anger when they appraise an artwork as contrary to their goals and values (e.g. in the case of Christians viewing artworks such as *Piss Christ* by Andres Serrano or *Virgin in a Condom* by Tania Kovats) and the result of deliberate action (e.g. when viewers think that the artist wanted to deliberately offend, insult or mock them). In turn, disgust stems from both an appraisal of goal incongruence (like in the case of anger) and appraising something as contaminated (i.e. dirty, harmful, unpleasant). Summing up, the negative components of emotions are also part of the aesthetic experience and it is worth broadening knowledge of the emotional experience of audiences faced with artworks which are controversial and may shock.

### Components of aesthetic emotional reactions: The non-verbalisation approach to measuring emotions

An aesthetic reaction to art should be considered in the context of existing theories concerning the components of emotional reactions to environmental stimuli [15–19] distinguishing three dimensions of affect that can be identified: namely valence, arousal, and dominance [17, 18]. Valence is defined as a degree of pleasantness/unpleasantness experienced while interacting with the object. Valence is the most intuitive of the dimensions when thinking about emotions [20], and thus the most commonly used dimension in aesthetic studies [21, 22]. Nevertheless, to fully describe an emotional reaction at least one additional dimension is needed, namely arousal [19]. Arousal can be defined as the state of an organism accompanying experiencing an emotion, ranging from sleep (or full calmness) to total excitement. Arousal describes the physical thrill of excitation that activates an organism and gives it the chance to react to the external event in a reasonably short period of time and with a reasonably large amount of force [23]. Arousal is often claimed to be disruptive for high order cognitive processes [24, 25], making it hard to think in a rational way. The last dimension found to be useful to describe affect [18] was dominance, which can be defined as an amount of power over or control exerted towards our emotional reactions [26, 27]. On the one hand, we can sometimes be dominated by emotional experiences and reactions, while in other situations we can also control the emotions experienced. Dominance was found to be quite closely correlated with valence [28, 29] in a way that negative emotions are perceived as uncontrollable (dominating over the individual), while positive are viewed as controllable.

Apart from valence, arousal and dominance, other dimensions could potentially be important for aesthetics, namely the origin of an affective reaction and its subjective significance [30, 31]. They were developed in the context of dual-mind theories distinguishing between uncontrolled (effortless or so-called automatic) and controlled (effortful or so-called reflective) ways of processing in the mind in the cognitive and emotional domains [15, 25, 30–33]. In line with this, the origin of an affective reaction can be defined as the engagement of automatic vs reflective mechanisms underlying the formation of emotional reactions. Automatic mechanisms do not require verbalisation to appear [33–37] and in the context of aesthetics, automatically originated emotions may be summarised as an immediate emotional reaction to the physical aspect of an artwork (colours, shapes etc.). Reflective emotional reactions are based on verbalised evaluative standards [33, 35, 38] that can lead to emotional reactions based on the interpretation of reality in the context of those standards [39]. In the context of aesthetics, reflectively originated emotions can be utilised with second-order reactions due to reflection and deliberation over the interpretation of the meaning of the piece of art. Some models of aesthetic experience focus on both these aspects of emotions, e.g the pleasure-interest model of aesthetic liking [40, 41], whereas others, e.g. the appraisal model of aesthetic experience, mainly explain a reflective (cognitive) aspect of aesthetic emotions [11–14]. A model of aesthetic appreciation and aesthetic judgements assumes the continuous development of changes in the affective state–from automatic to deliberate affective evaluation [2]. Both automatic and reflective processes may be present when perceiving and reacting to art (and especially critical art). First of all, shape, colour, form etc. are experienced while viewing artwork and evoke direct, primary emotions. Round shapes are generally safer, thus more positively evaluated (e.g. [42]), than sharp edges at the automatic level of evaluation. The automatic level of evaluation may also be susceptible to more complex features of the stimulus. For instance, cruelty presented in a figurative artwork may be automatically interpreted through the emotional facial expressions of figures (i.e. fear) or through biologically meaningful stimuli (like fresh blood, injury etc.). But looking at the art also engages intellectual mechanisms of interpreting the visual features of the artwork as well as searching for the sense of the art. This triggers a reflective reappraisal. The sharp edge is desirable when you are intending to cut something and finding a lost knife in this context is quite joyful. In the case of critical art, the same occurs when something which is, for example, revealing may be interpreted as an important symbol of a positively evaluated idea, thus the aesthetic reaction to the artwork may be, paradoxically, generally positive [43, 44].

Subjective significance is postulated to be the reflective form of activation that is analogous to arousal [28, 31], which can be defined as a form of attitude towards the situation, stating its importance and significance for the realisation of one’s aims and goals. In other words, we can say that subjective significance is the factor underlying perseverance in the processing of effortful, reflective, mental processes [31], which can explain why people engage in such processing instead of relying on cognitive shortcuts [45, 46]. In the context of the perception of art and the understanding of the meaning of artwork, subjective significance may be the factor responsible for engagement in the process of decoding art and getting a sense of the message sent by the author. A more subjectively significant experience seems to be that the more cognitive effort is put into understanding the artwork, the more elaborate (and thus more reflective in origin) the aesthetic emotions that may appear. Similar effects to those predicted by subjective significance are present in the effortless theory of flow and attention [43, 44]. When we interpret a situation as congruent to our interests and goals, even effortful processing may seem to be effortless from the subjective point of view. This does not mean effortless processing in terms of cognitive processes, but more cognitive resources available and less fatigue associated with a certain situation.

For the operationalisation of the theories discussed so far, we used the Self-Assessment Manikin (SAM) scales [17], originally developed for valence, arousal and dominance, in order to provide a pictorial representation of an affect. Thanks to this, we achieved a reduction of interference between experiencing an emotion and assessing its state [17, 32]. The SAMs are made up of a series of human figures expressing discreet changes in their bodily responses which are characteristic of emotions. For example, valence starts from a schematic face expressing sadness while it ends with a smiling schematic face. It may be considered as problematic to use the SAM for valence because it does not directly measure the pleasantness of a response without referring to basic categorical emotions such as sadness or joy (c.f. the instructions used in the current experiment), which may somehow be misleading when following a dimensional response to emotion. Nevertheless, when the number of labels for specific emotions presented to the participant is large (as in the case of the current study for negative SAM in which the states described as panic, irritation, disgust, despair, defeat, or crisis were listed; as compared to positive SAM: fun, delight, happiness, relaxation, satisfaction or recreation), we expected a prototypical emotional reaction to be formed in the mind of participants. Arousal SAM uses the metaphor of a thrill in the body, leading to an explosion of excitement, while dominance is expressed by the size of an individual, starting from a figure that is dominated and ending with a figure dominating over space. SAM scales were used to assess the affective norms for different types of stimuli, including pictures, words, and sentences, each time showing the reliability of measurement and stability of assessment over time [26, 28, 29, 36, 47, 48].

In order to measure origin and subjective significance, two new SAM scales were introduced [32]. The concept of origin was built upon a metaphor of the heart vs. mind dichotomy, widely distributed in western culture, contrasting the reasons of the heart to the reasons of the mind [49]. Reasons of the heart are treated as automatic and immediate reactions that are hard to resist, while reasons of the mind are deliberated upon, delayed and rational. The same distinction fits the difference between the automatic and reflective origins of an emotional reaction [32, 33]. The subjective significance scale was based on an abstract representation of the importance of the experience, expressed as an exclamation mark provoking careful consideration.

The studies for verbal materials [28, 32, 50], as well as music pieces [15], showed that the reliability (including split-half analyses as well as the stability of assessments) of origin and subjective significance SAM scales are comparable to the other SAM scales. All five SAM scales described above appeared to be useful tools for assessing aesthetic reactions to music and art [15, 51, 52].

### Aesthetic judgement: Understanding and liking

In experimental aesthetics, some of the most frequently examined aesthetic judgements were those of self-rated understanding (i.e. subjective understanding, sense of understanding) and/or liking [e.g. 51, 53–58]. In line with a model of aesthetic appreciation and aesthetic judgements [2], liking may be related to either output of art processing–emotion or judgements. Liking may result from automatic bottom-up processing, in which case it should be treated as an aspect of emotional output. On the contrary, when liking is connected with the cognitive-based way of reception of art, it should be treated as an aesthetic judgement. This way of processing art might be provoked experimentally, for example by asking participants to assess the artwork’s subjective understanding. They will then be forced to engage in the process of cognitive mastering (i.e. an attempt to give an interpretation) and evaluate its effects. If the subjects are then asked about liking, they will make another aesthetic judgement. In the present study, we adopted this approach by first asking people to rate their understanding and then their liking of the artworks. As a consequence, we treat aesthetic judgements in terms of understanding and liking.

The importance of understanding art for the recognition of its value and to appreciate it is not only a concern in relation to controversial art, but also in relation to contemporary art in general. Contemporary art is a cognitive challenge, especially for viewers who are not experts. The artworks that are currently being created are often in a form distant from conventional art and are perceived as bizarre and not pleasing to the eye. Several psychological models of viewers’ responses to such art emphasise that a feeling of a satisfactory "solution" to the cognitive puzzle of interpreting a contemporary artwork is self-rewarding and gives the viewers pleasure [1, 2, 59–61] (see also the hypothesis of effort after meaning and hedonic value [62] or the concept of the aesthetic “Aha!” [63]). Therefore, at the level of structural analysis (regardless of the topic of a given artwork), non-professionals might already feel uncomfortable due to a lack of understanding and judge a challenging artwork unfavourably (however, some research has shown that non-expert observers may also actively evaluate abstract artworks [64]). In other words, the abovementioned theories underline the link between a sense of understanding and aesthetic enjoyment and/or liking. Subjective understanding, and consequently liking of contemporary artworks may result both from attempts to interpret a given contemporary artwork on one’s own, thanks to engagement in the reception of this artwork (which is more probable in the case of non-experts who are more open to experiences [65] and are characterised by a low need for closure [58]), and from getting acquainted with contextual information concerning artworks (e.g. descriptions that are made available by galleries as part of an exhibition).

### Reception of artworks in an art gallery and a laboratory setting

Visual art affects the audience more intensely when viewed in the situational context of a gallery/museum than when seen in another situational context [66, 67]; however, the influence of the situational context of presenting art on aesthetic experience may be moderated by an artwork’s genre (e.g. street art is not less appreciated in the street than in a museum context [56, 68]). It seems that typical exhibition space motivates an audience to contemplate artworks as it has been shown that artworks are both viewed for longer and evaluated to be more interesting in a museum than in a laboratory context [55].

The situational context of an art gallery increases both the intensity of the aesthetic affective experience [51, 54, 69–71], the hedonic value and the appreciation of the artworks [51, 54, 55, 57, 69–72]. In a study in which aesthetic emotions were assessed on SAM scales [51], it was found that participants who viewed installation art in a contemporary art gallery were more positively aroused, the source of their emotions was to a greater extent automatic than reflective, and their emotions were considered as more important than those which viewers felt in a laboratory setting. Moreover, Brieber and colleagues [54] showed that presenting art in a gallery facilitates understanding–but the effect of the situational context of the exhibition space on subjective (self-rated) understanding was not replicated in other studies [51, 53, 55, 57].

### Contextual information as interpretative hints changes the aesthetic experience

The types of contextual information that can facilitate making sense of art are titles [62, 73–75] (see also [76–78]), extended descriptions of artworks [51, 62, 79, 80] (see also [81]), and curatorial tours [82, 83]. However, understanding seems to be enhanced more with extended descriptions than the titles themselves [51, 79] (see also [80]).

To the best of our knowledge, the influence of the method of providing contextual information on the aesthetic experience has only been tested in one piece of research to date [84]. In that study, carried out in a laboratory setting, contextual information (a fragment of the original curatorial description) was delivered visually (participants read it) before an artwork was presented, was listened to before or while viewing a painting, or was not given at all (the control condition). Contemporary paintings evoked more positive emotions and were liked more when they were viewed while listening to the contextual information than after listening to catalogue descriptions, which was explained against the background of the model of working memory [85] and the concept according to which processing fluency increases aesthetic pleasure [86]. Aesthetic fluency decreased when participants had to maintain information that they had listened to in working memory [84] (see also [87], for linking working memory, cognitive load, and aesthetic fluency).

The closest situation to the typical receiving of an artwork seems to be getting acquainted with the description, either by reading it or by listening to it, while viewing the artwork. Labels are usually available next to artworks in galleries and art albums. In addition, during a curatorial tour in the exhibition space, which sometimes also used to be available to an audience as a movie, we view the artworks and at the same time listen to what the guide has to say about them. In our study, we wanted to explore whether any of these ways of presenting a curatorial description simultaneously with the artworks, i.e. giving written text to be read or to be listened to, has a more significant impact on aesthetic emotions and judgements. Reading, compared to listening to the text, engages working memory to a greater extent and requires more cognitive capacity. While listening, the text is processed by the auditory channel (more specifically–the phonological loop) in working memory. During reading however (provided that a reader is proficient in this activity), the text is processed by two channels, visual (in the visuospatial sketchpad) and auditory [85, 88]. On the other hand, whereas listening to the text facilitates a focus on the gist and results in better overall understanding, self-controlled reading leads to a concentration on the detailed information and should be beneficial when recalling the text [89, 90].

At the same time, the representation of the language semantics in the brain is independent of whether meaning is extracted from spoken or written words [91]. Therefore, engagement in the processing of artworks (i.e. the cognitive mastering process–the deliberate process of interpretation of an artwork [2]) can be greater both when the viewers become involved in reading the curatorial description and when they listen to the curatorial information. Regardless of the communication channel, providing contextual information as curatorial descriptions ensures the delivery of interpretative hints, which may support the process of meaning-making in the situation of viewing challenging contemporary art.

### The present study

When considering aesthetic emotions, we expected the gallery context to increase the intensity of emotional reactions to artworks in comparison with the context of looking at the art in the laboratory setting [2, 51, 54, 55, 57, 69–72]. More specifically, we expected the valence (H1), dominance (H2), arousal (H3) and subjective significance (H4) to be higher, while we expected assessments of the origin of an emotional reaction to be different between conditions (without a specific direction of differences) (H5) in comparison with the laboratory setting context. Taking into account the contextual information, we expected that reading the curatorial information by oneself, as most engaging the working memory and the most demanding process [85, 88, 89], would increase the intensity of emotional reactions to artworks in comparison with the condition of no contextual information and with the condition of listening to the contextual information. We expected the valence [86] (H6), dominance (H7), arousal (H8) and subjective significance (H9) to be higher, and the origin to be different between conditions (without a specific direction of differences) (H10) in the reading condition compared to the no contextual information and the listening to contextual information conditions. Considering aesthetic judgement, in terms of liking, we expected artworks to be liked more in the gallery context than in the context of the laboratory setting (H11) [2, 51, 54, 55, 57]. Moreover, we hypothesised that subjective understanding is higher when viewers are provided with extended contextual information (namely–curatorial information) in written form [62, 79] or read out loud form [51, 80] in comparison with viewing artworks without any contextual information (H12) [2].

Some of our predictions concerned the replication of the previous findings, namely: H1 [51, 54, 69–71], H3 [51, 54], H4 [48], H5 [48], H11 [51, 53, 55, 57], and H12 [51, 62, 79, 80]. However, we tested these hypotheses in a particular situation of viewing critical art by Polish young adults (who are more conservative than the majority of citizens in Europe and the Western world–see e.g. [5] and S1 Appendix: curatorial information on Artwork 5). Others were original–they aimed to test new hypotheses (H2, H6, H7, H8, H9, H10).

## Materials and methods

### Participants

One hundred and twenty volunteer students of non-artistic MA courses participated in the study (81 females, all participants aged between 19 and 27 years, *M*_age_ = 21.2, *SD*_age_ = 1.4). Based on the previous study of Szubielska et al. [51], we expected the η_p_^2^ to range from.09 to.3 regarding emotional experiences and from.09 to.16 regarding aesthetic judgements. Considering the lowest possible of these values (η_p_^2^ = .09), we conducted a-priori power analyses using the G-Power 3.1 software [92] before the current study, which showed that to achieve a high power of.8 we would need at least 82 participants for the simple effects and least 115 participants for the interaction of two factors.

We controlled participants’ self-reported expertise in the field of art (*M* = 1.65, *SD* = 1.49; a range of scores: 0–7) and interest in contemporary art (*M* = 2.00, *SD* = 1.63; a range of scores: 0–7) on 8-point scales (the ends of the scales were described as follows: 0 = *not at all*, 7 = *extremely*). The study was conducted in accordance with the Helsinki Declaration and approved by the Ethics Committee of the Institute of Psychology of the John Paul II Catholic University of Lublin. The participants’ informed written consent was obtained.

### Design

The study was conducted following a between-subjects design, there were two main factors that were manipulated: context of viewing (2 levels: art-gallery vs laboratory setting) and contextual information provided (3 levels: no contextual information vs reading vs listening to contextual information), thus participants were assigned to six experimental conditions: (1) in the art gallery, lack of curatorial information, (2) in the art gallery, curatorial information read on one’s own, (3) in the art gallery, curatorial information listened to, (4) in the laboratory setting, lack of curatorial information, (5) in the laboratory setting, curatorial information read on one’s own, (6) in the laboratory setting, curatorial information listened to. All experimental groups were provided with an equal number of participants (all *n*s = 20). The dependent variables were aesthetic emotions and aesthetic judgements.

### Material

The artworks we used in the current study (together with their curator’s description, see S1 Appendix) were presented at the open call part of the *Three plagues* exhibition (curated by Agnieszka Cieślak and Magdalena Linkowska) at the Galeria Labirynt art gallery in Lublin (Poland) from 01 September 2019 until 11 November 2019. Only young, relatively unknown artists took part in this part of the exhibition. For organisational reasons, we included in the study those eleven artworks that did not have a soundtrack presented through headphones (three works were excluded). The exhibition’s topic concerned the "plagues" of nationalism, racism, and religious fundamentalism–which characterise an aggressive, all-powerful, totally irrational way that people (and societies) think and behave. In the works presented (quite formally complex installations, videos of objects, and a series of photographs–see S1 Appendix for each artwork’s medium of expression), the artists addressed this topic in a critical manner, manifesting anxiety, disagreement or criticism of reality and people who accept violence.

### Procedure

In each of the six experimental conditions, the study was conducted in groups of about five participants and the answers were collected by a self-administered paper-and-pencil questionnaire. The participants were asked to answer on their own (i.e. without consulting other individuals in the group and without commenting on the artworks that they viewed). The artworks were viewed in a fixed order (see S1 Appendix) on the premises of the art gallery (original artworks) at a specifically scheduled time, or in a laboratory setting context (video recording of the work presented on the wall using a multimedia projector and taking the form of a movie in a loop; we used eleven movie excerpts–one excerpt per artwork, each looped). The artworks were watched without providing contextual information (the labels in the exhibition space were covered for the duration of the study) or the viewing was accompanied by getting acquainted with the curatorial information. The curatorial information was either read independently by each participant on their own, or the participants listened to the information (the experimenter read it aloud). Participants rated, in a fixed order, the emotions evoked by each artwork on five 9-point Likert SAM scales (after having previously been familiarised with the instructions for these scales): valence, arousal, dominance, origin, and subjective significance [28] and their aesthetic judgements, namely to what extent they subjectively understand and like artworks on 8-point Likert scales (with scales running from 0 to 7 corresponding to *not at all*, and *extremely*). Thanks to the adoption of such an order of scales (i.e. first asking for an assessment of emotional reactions, then a cognitively based aesthetic judgement), we analyse aesthetic judgements which are based on emotions (the opposite order would have led to biases based on a cognitive interpretation of the emotional reaction i.e. it would probably enhance the interpretation of emotions as more reflectively originated). The amount of time for viewing and assessing each artwork was not limited (but all participants in a particular test group had to evaluate the particular artwork before they moved on to the next one). After viewing and evaluating all the works included in the experiments, without warning a memory test was sprung upon all the groups which had been acquainted with the curatorial descriptions. The test consisted of 20 questions on the content found in the curatorial descriptions. Four alternative answers were provided for each single-choice question (see S2 Appendix).

## Results

### Preliminary analyses

**Fig 1:**
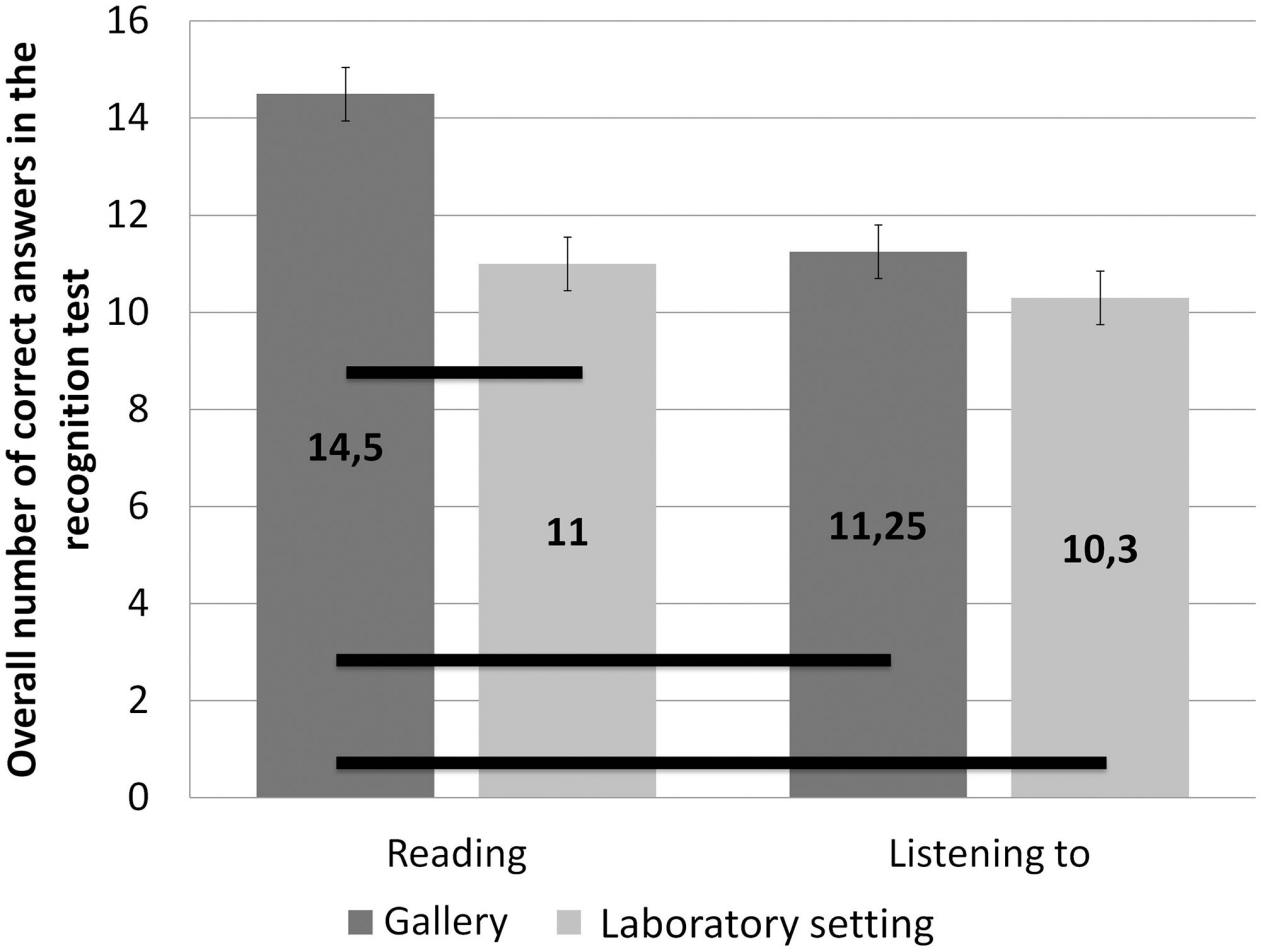
An interaction effect between the way of providing contextual information and the situational context on the memory of curatorial information. Error bars represent +/- 1 standard error.

We analysed whether the situational context of the reception of the art and the way in which the curatorial information was provided had influenced the amount of content included in the descriptions that was remembered. We conducted a two-way analysis of variance (ANOVA) for between-subjects independent variables of the situational context of presentation of the art (in the art gallery, in the laboratory setting) and the way of providing the contextual information (reading it on one’s own, listening to it) with the dependent variable being the overall number of correct answers in the recognition test. The analysis showed the main effects of (i) the situational context: *F*(1,76) = 16.18, *p* < .001, η_p_^2^ = .18, as participants recognised more information correctly in the gallery (*M* = 12.88, *SEM* = 0.39) than in the laboratory setting context (*M* = 10.65, *SEM* = 0.39) and (ii) the way of providing contextual information: *F*(1,76) = 12.75, *p* = .001, η_p_^2^ = .14, as participants recognised more pieces of information correctly when reading them on their own (*M* = 12.75, *SEM* = 0.39) than when listening to them (*M* = 10.78, *SEM* = 0.39), and, in addition, showed the two-way interaction of the situational context and the way of providing contextual information: *F*(1,76) = 5.31, *p* = .024, *η*^*2*^ = .07. Follow-up comparisons of the interaction effect by post hoc analyses using Bonferroni adjustments (here and throughout) revealed that participants who were tested in the gallery condition and read curatorial descriptions on their own remembered more information than both those who were tested in the gallery condition and listened to the curatorial descriptions (*p* < .001), those who were tested in the laboratory setting and read the curatorial information on their own (*p* < .001), and those who were tested in the laboratory setting and listened to the curatorial descriptions (*p* < .001) (see Fig 1). Other post hoc comparisons did not show significant differences between the conditions (all *p*s >.228).

### Aesthetic emotions

In order to validate the hypotheses, we conducted five two-way independent ANOVAs for between-subjects independent variables of the situational context of presentation of the art (2) and the way of providing the contextual information (3) with the dependent variable being respectively: valence, dominance, arousal, subjective significance, origin (see Table 2). For each ANOVA, we computed a simple orthogonal planned contrast analysis (one of the standard contrasts available in SPSS) to test hypotheses 6–10. In the case of significant interaction between the situational context and contextual information, we conducted follow-up comparisons of the interaction effect by post hoc analyses.

#### Valence

The hypothesis that states that valence is higher (i.e., less negative–see Table 1) in the context of the gallery in comparison with that of the laboratory setting (H1) was confirmed (see Table 2). Participants felt fewer negative emotions in the gallery situational context (*M* = 4.59, *SEM* = 0.13) than in the laboratory setting situational context (*M* = 4.06, *SEM* = 0.13). We expected the valence to be higher in the reading condition compared to the no contextual information and the listening to contextual information conditions (H6). This hypothesis was not confirmed by the contrast analysis: *F*(2, 114) = 2.21, *p* = .114, η_p_^2^ = .04.

**Table 1:** Means and standard deviations (presented in parentheses) of aesthetic emotion on the dimensions of valence, arousal, dominance, origin, subjective significance, and of aesthetic judgement on the dimensions of understanding and liking in each group.

	Gallery context			Laboratory setting context		
	N	R	L	N	R	L
*Emotion*						
Valence	4.65 (0.92)	4.57 (0.62)	4.55 (0.95)	4.20 (1.02)	4.40 (1.12)	3.58 (1.10)
Arousal	4.74 (1.08)	4.92 (0.83)	5.18 (0.99)	4.20 (1.14)	4.73 (1.17)	3.93 (1.03)
Dominance	5.26 (1.14)	4.64 (0.83)	4.44 (1.03)	4.11 (0.83)	4.95 (1.15)	3.78 (0.89)
Origin	5.26 (1.21)	5.36 (0.77)	5.24 (0.78)	4.40 (0.90)	5.57 (1.17)	4.96 (0.73)
Significance	5.20 (1.37)	5.71 (0.68)	5.67 (1.11)	3.89 (1.05)	5.14 (1.17)	4.01 (0.75)
*Judgement*						
Understanding	3.62 (1.45)	3.97 (0.76)	4.25 (0.87)	2.93 (1.47)	4.58 (0.86)	3.43 (1.46)
Liking	3.73 (1.41)	3.93 (0.78)	4.04 (1.21)	3.07 (0.93)	3.47 (1.11)	2.12 (1.42)

*Note*. N = no contextual information; R = reading contextual information; L = listening to contextual information.

**Table 2:** Effects of situational context and the way of providing contextual information on aesthetic emotion and aesthetic judgement: Inferential statistics.

	Situational context	Contextual information	Two-way interaction
*Emotion*			
Valence	*F*(1, 114) = 9.04, *p* = .003, η_p_^2^ = .07	*F*(2, 114) = 2.21, *p* = .114, η_p_^2^ = .04	*F*(2, 114) = 1.80, *p* = .171, η_p_^2^ = .03
Arousal	*F*(1, 114) = 12.01, *p* = .001, η_p_^2^ = .10	*F*(2, 114) = 1.24, *p* = .293, η_p_^2^ = .02	*F*(2, 114) = 2.67, *p* = .074, η_p_^2^ = .05
Dominance	*F*(1, 114) = 7.66, *p* = .007, η_p_^2^ = .06	*F*(2, 114) = 5.62, *p* = .005, η_p_^2^ = .09	*F*(2, 114) = 5.70, *p* = .004, η_p_^2^ = .09
Origin	*F*(1, 114) = 3.20, *p* = .076, η_p_^2^ = .03	*F*(2, 114) = 4.59, *p* = .012, η_p_^2^ = .008	*F*(2, 114) = 3.20, *p* = .045, η_p_^2^ = .05
Significance	*F*(1, 114) = 37.71, *p* < .001, η_p_^2^ = .25	*F*(2, 114) = 7.32, *p* = .001, η_p_^2^ = .11	*F*(2, 114) = 2.80, *p* = .065, η_p_^2^ = .05
*Judgement*			
Understanding	*F*(1, 114) = 1.95, *p* = .166, η_p_^2^ = .02	*F*(2, 114) = 7.15, *p* = .001, η_p_^2^ = .11	*F*(2, 114) = 4.40, *p* = .014, η_p_^2^ = .07
Liking	*F*(1, 114) = 22.62, *p* < .001, η_p_^2^ = .17	*F*(2, 114) = 2.81, *p* = .064, η_p_^2^ = .05	*F*(2, 114) = 4.56, *p* = .012, η_p_^2^ = .07

#### Dominance

The hypothesis according to which dominance is higher in the gallery condition than in the laboratory setting condition (H2) was confirmed (see Table 2), since in the condition in which the artworks were seen in the gallery, they evoked more dominating emotional reactions (*M* = 4.78, *SEM* = 0.13) than in the laboratory setting condition (*M* = 4.28, *SEM* = 0.13). We expected that reading the information by oneself increases the dominance (control over emotional reactions) when compared to the condition of no contextual information and to the condition of listening to the contextual information (H7). This hypothesis was confirmed by the contrast analysis: *F*(2, 114) = 5.62, *p* = .005, η_p_^2^ = .09, since dominance was higher in the reading condition (*M* = 4.79, *SEM* = 0.17) than both in the listening to condition (*M* = 4.11, *SEM* = 0.17) and the no contextual information condition (*M* = 4.69, *SEM* = 0.17). Furthermore, the interaction between the situational context and contextual information was significant (see Table 2). Post hoc analyses yielded the conclusion that participants who listened to the curatorial descriptions and were tested in the gallery condition rated dominance as higher than those who listened to the curatorial descriptions and were tested in the laboratory setting (*p* = .037). Similarly, higher ratings of dominance were revealed in the gallery condition without contextual information than in the laboratory setting condition without contextual information (*p* < .001). Moreover, in the gallery condition dominance was higher in the participants who did not obtain contextual information than in participants who listened to contextual information (*p* = .027). In the case of participants who looked at artworks in the laboratory setting, dominance was rated as higher by participants who read curatorial information in comparison with both those who listened to contextual information (*p* = .001) and those who did not obtain contextual information (*p* = .025) (see Table 1). Other post hoc comparisons did not show significant differences between the conditions (all *p*s >.140).

#### Arousal

The hypothesis that arousal is higher in the gallery condition than in the laboratory setting condition (H3) was also confirmed (see Table 2). In line with our prediction, participants were more aroused in the gallery context (*M* = 4.95, *SEM* = 0.14) than in the laboratory setting context (*M* = 4.29, *SEM* = 0.14). The hypothesis according to which arousal is higher in the reading condition compared to the no contextual information and the listening to contextual information conditions (H8) was not confirmed since the analysis of contrasts did not show a significant effect: *F*(2, 114) = 1.24, *p* = .293, η_p_^2^ = .02.

#### Subjective significance

Another hypothesis that was confirmed was that concerning the subjective significance of emotions felt in the gallery situational context in comparison with those felt in the laboratory setting situational context (H4) (see Table 2). As predicted, artworks seen in the gallery evoked more subjectively significant emotional reactions (*M* = 5.52, *SEM* = 0.14), than those seen in the laboratory setting (*M* = 4.35, *SEM* = 0.14). The hypothesis that reading the curatorial information increases the subjective significance of emotions in comparison with the condition of no contextual information and with the condition of listening to contextual information (H9) was confirmed by the significant result of the contrast analysis: *F*(2, 114) = 7.32, *p* = .001, η_p_^2^ = .11. In line with our predictions, participants rated subjective significance higher in the reading condition (*M* = 5.43, *SEM* = 0.19) than in both the listening to condition (*M* = 4.84, *SEM* = 0.19) and the no contextual information condition (*M* = 4.54, *SEM* = 0.19).

#### Origin

The hypothesis that the origin of assessments of an emotional reaction differed between the situational context of the gallery and that in the laboratory setting (H5) was not confirmed (see Table 2). However, in the gallery condition emotions were rather reflective (*M* = 5.29, *SEM* = 0.13) and in the laboratory setting condition emotions were rather automatic (*M* = 4.98, *SEM* = 0.13). The contrast analysis testing the hypothesis that the origin of assessments of an emotional reaction on reading the curatorial information condition differed between conditions of not being familiar with contextual information and listening to the contextual information (H10) was statistically significant: *F*(2, 114) = 4.59, *p* = .012, η_p_^2^ = .08, which confirmed our expectations. Origin was rated as more reflective in the reading condition (*M* = 5.47, *SEM* = 0.15) than in both the listening to condition (*M* = 5.10, *SEM* = 0.15) and the no contextual information condition (*M* = 4.83, *SEM* = 0.15). Moreover, the interaction between the situational context and contextual information was significant (see Table 2). Post hoc analyses showed that in the laboratory setting origin was lower (i.e. more automatic–see Table 1) when participants were not familiar with the contextual information than when participants read the curatorial information (*p* < .001). Moreover, when participants were not provided with contextual information origin was higher (i.e. more reflective–see Table 1) in the gallery condition than in the laboratory setting condition (*p* = .005). Other post hoc comparisons did not show significant differences between conditions (all *p*s >.132).

### Aesthetic judgements

Analysing aesthetic judgement, we took an analogical approach as when analysing aesthetic emotions and performed two two-way ANOVAs with liking or understanding as the dependent variables.

#### Liking

We expected that artworks are liked more in the situational context of a gallery than in that of a laboratory setting (H11). The hypothesis was confirmed (see Table 2) as liking was higher in the gallery context (*M* = 3.90, *SEM* = 0.14) than in the laboratory setting context (*M* = 2.89, *SEM* = 0.17). The main effect of contextual information was not significant, but the interaction between the context of presenting the art and contextual information reached significance (see Table 2). Post hoc analyses of this interaction showed that in the condition of listening to contextual information, art was liked more when viewed in the gallery context than in that of the laboratory setting (*p* < .001). In the condition of viewing art in the laboratory setting, artworks were liked more when participants read the curatorial information on their own compared to both when participants listened to the curatorial information (*p* = .001) or when they were not familiar with the contextual information (*p* = .035) (see Table 1). Other post hoc comparisons did not show significant differences between conditions (all *p*s >.074).

#### Understanding

As we expected, providing both written and read-out-loud curatorial information increases subjective understanding in comparison with the control condition of not providing curatorial descriptions (H12), since the contrast analysis was significant: *F*(2, 114) = 7.15, *p* = .001, η_p_^2^ = .11. Subjective understanding was higher both when viewers were provided with written curatorial information (*M* = 4.28, *SEM* = 0.14) and when information was read out loud by an experimenter (*M* = 3.84, *SEM* = 0.20) when compared to viewing artworks without any contextual information (*M* = 3.27, *SEM* = 0.23), which confirms our hypothesis. The main effect of situational context was not significant, but the interaction between the factors which were analysed was significant (see Table 2). Follow-up comparisons of this interaction showed that in the condition of listening to contextual information, artworks were better understood when viewed in the context of the gallery than in that of the laboratory setting (*p* = .031). In the condition of viewing art in the laboratory setting, subjective understanding was higher in participants who read the curatorial information on their own than in both participants who listened to the curatorial information (*p* = .009) and participants who were not provided with any contextual information (*p* < .001) (see Table 1). Other post hoc comparisons did not show significant differences between conditions (all *p*s >.069).

## Discussion

### The influence of the situational context of presenting artworks and the way of providing curatorial information on emotional experience

Taking into account the emotional reactions to the artworks presented we showed that an art gallery context increases the observer’s experience of aesthetic emotions in terms of valence (H1 was confirmed). The emotional reactions to artworks were less negative in the gallery than in the laboratory setting, which is generally in line with earlier studies [51, 54, 69–71].

Considering the dominance dimension of affect, representing the control of emotional experiences: uncontrollable (lower values) vs under control (higher values), we expected also that artworks would evoke more dominant emotional reactions in the gallery than in the laboratory setting (H2). Once again, a gallery effect was found and the hypothesis was confirmed. Emotional reactions were perceived as more controllable in the gallery context (these effects were significant in conditions of no contextual information provided and in listening to the curator’s information, but not in the case of reading it). This effect may be interpreted in the context of the relationship between valence and dominance: i.e. positive emotions are typically perceived as controllable, while negative ones were perceived as dominating our experiences [15, 17, 26–28]. The dominance effect is therefore congruent with the valence effect of the current study, showing more positive assessments for the gallery context.

Taking into account the activational aspects of emotional reactions (arousal (H3) and subjective significance (H4)), situational context appeared, once again, to be an important factor: in the gallery the emotions experienced were more arousing and more subjectively significant, which is in line with our hypotheses and a previous study using SAM scales to measure aesthetic arousal and subjective significance [51]. Finally, for origin we expected differences between two different situational contexts (H5), but no such effect was found. Interestingly, the post hoc analysis for an interaction effect in the origin scale showed that when no curatorial information was provided, the origin was more reflective in the gallery than in a laboratory setting. This suggest that the gallery context provokes more deliberation over the art perceived. Putting all the results together, they suggest the existence of a gallery effect [51, 54, 55, 57, 67, 69–72, 93], i.e. artworks in a gallery evoke more intense emotional reactions than the same artworks presented in the laboratory setting.

Another interesting aspect of the study was the investigation into how the way of providing curatorial information influences emotional experiences. For valence, no effect of contextual information was found (H6 was not confirmed). This is not surprising, since valence, as the most intuitive dimension ascribing emotions [16, 20, 33, 94], does not require additional information to appear. In the context of dual-process theories [30, 95], valence is thought to represent a so-called simplified procession of the experiential mind (or System 1).

We expected that reading the information by oneself would increase dominance (control over emotional reactions) in comparison with the condition of no contextual information and with the condition of listening to the contextual information (H7), which was confirmed by the results obtained (particularly when participants were tested in the laboratory setting). Also, interaction was found between context and contextual information, showing that in the reading condition the gallery effect disappeared, i.e. emotional experiences were perceived as comparably controlled in the gallery and in the laboratory setting. This is an interesting effect showing, to some extent, that the cognitive effort needed to read and understand the contextual information may shape the way we perceive control over aesthetic emotions.

It is interesting that reading contextual information influenced the origin of an assessment of affect (H10 was confirmed). The written contextual information made the aesthetic emotions experienced by individuals more reflective in comparison with those experienced with a lack of contextual information or listened to contextual information. The reading process engages the cognitive effort and thus processing in a rational mind [30, 31, 95, 96] which results in more reflectively originated emotional reactions. We also found that in a laboratory setting, no curatorial information provided led to more automatic originated reactions in comparison with reading information conditions. When no context is provided, participants may mostly react to the perceptual features, but not so obviously get the message of the art.

Taking into account the activational aspects of emotional reactions (arousal H8 and subjective significance H9), we predicted that participants reactions would be more intense (higher) in the reading condition compared to the no contextual information and the listening to contextual information conditions. We found no such effects for arousal (thus H8 was not confirmed). Considering subjective significance, hypothesis (H9) was confirmed. The condition of reading contextual information resulted in more subjectively significant aesthetic emotional reactions in comparison with both listening to and no contextual information provided. The reasoning presented for the origin effect is once again valid, since subjective significance, by definition [31], and measurement [28, 32] are forms of reflective activation specific to the rational mind and effortful processing [30].

### The influence of the situational context and the way of providing curatorial information on aesthetic judgement

Considering aesthetic judgement, we formulated two hypotheses which were intended to replicate the previous findings on the effect of situational context on liking (H11) [51, 54, 55, 57] and providing extended contextual information on the subjective understanding of contemporary artworks (H12) [51, 62, 79, 80]. In the current study, we aimed to test if those results would replicate with regard to critical visual art.

In line with earlier studies in the field [51, 54, 55, 57] and our hypothesis (H11), artworks were liked more in the gallery than in the laboratory setting. This effect was particularly noticeable when participants listened to curatorial information. Bearing in mind that the stimuli used in the current study were exemplars of contemporary critical art that addresses controversial topics, it is understandable that the ratings for liking were rather low [11, 13]–especially in the laboratory setting, as the aesthetic experience of art is weakened in the laboratory setting compared to an exhibition space [51, 54, 57, 66, 67, 69–72].

As predicted (H12), subjective understanding was higher both when viewers read and when they listened to curatorial information in comparison to viewing artworks without any contextual information. This result is in line with other studies in which extended contextual information supported the understanding of artworks [51, 62, 80]. Moreover, our study showed that in the laboratory setting self-rated understanding was higher when participants read the curatorial descriptions on their own rather than when they listened to them. At the same time, in the context of the laboratory setting, self-controlled reading did not lead to better memorising of detailed information from curatorial descriptions in comparison with the condition of listening to curatorial information (which does not confirm previous research showing that reading contributes more than listening to remembering detailed information [89, 90]).

In the previous study on the memory of paintings, it was found that artworks that were memorised better were rated as more pleasant and beautiful [97]. Furthermore, in another study contemporary artworks (paintings, photographs and collages) were better remembered, rated as more interesting, and more liked in the museum than in the laboratory [54]. In turn, our study shows that a higher appreciation of art is not related to remembering the contextual information listened to (for further exploration of this issue see [72]). The participants who listened to contextual information within the gallery context and in the laboratory setting did not differ in the amount of information coming from curatorial descriptions that they recognised, and at the same time artworks were better liked (and better understood) after listening to curatorial information in the gallery than in the laboratory setting. Similarly, in the condition of presenting pieces of art in the laboratory setting, memory of curatorial descriptions did not differ depending on the way they were presented, and aesthetic liking (and understanding) was higher when participants read the curatorial information than when contextual information was listened to (or was not provided at all). Moreover, in the condition of reading the curatorial description of the artworks, more information was remembered in the gallery than in the laboratory setting (which may mean that in the conditions of the gallery, the viewers read the curatorial descriptions more carefully), and aesthetic liking did not differ due to the situational context of the presentation. Also, in the condition of viewing artworks in the gallery, more information was recognised by participants who were reading than those who listened to curatorial descriptions (which is in line with earlier studies [88, 89]), but the liking of artworks did not differ due to the way of providing contextual information. To sum up, in the case of critical art, which raises topics that are unpleasant for many viewers, gaining detailed knowledge about a given work probably does not translate into liking it since the curatorial information stresses the strands which are inconvenient for society. In other words, if the viewers appraise values manifested by the given artwork as inconsistent with their own values or goals (and an artist’s values become more evident to viewers who are familiar with curatorial descriptions), they can experience negative, hostile emotions and negate artworks’ value [11**–**14].

### Limitations

The current study should be interpreted in the light of some limitations. The first limitation concerns the lack of dissociation of the role of situational context and genuineness (for further exploration of this issue cf. [53] vs [57]), however in a similar manner to our procedure, in most studies in this field genuine artworks were shown in the gallery/museum context, while video/photo documentation of pieces of art was presented in a laboratory setting [51, 54, 55, 69–72]. Second, participants were tested in small groups of people (similar limitations apply to previous studies conducted in an art gallery (e.g. [5, 51, 80, 82, 83]). Although participants were asked not to communicate with each other when giving answers and not to express their opinions on artworks loudly, testing in groups could influence the responses given. Non-verbal reactions could be evidence of, for example, interest in a given artwork (approaching it and watching it carefully in gallery conditions) or rejection of a particular piece of art (hostile facial expression). Observing such behavioural cues of other viewers’ opinions may have modified the responses of some participants (i.e. those more susceptible to group influence). Possibly the results would be slightly different if the participants were tested individually. Third, the initial structural properties of the item being evaluated (e.g. colour, symmetry) may influence the emotional reaction (for a review of the literature, see [1]), especially in terms of valence. Nevertheless, in the case of the relatively complex artworks that we showed to the participants (films, series of photographs, or installations or objects consisting of many elements) it was difficult to control how the structural features of the stimuli change emotions. Additionally, the matter was complicated by the fact that in the situational context of the gallery, the participants approached a given artwork that they rated, but at the same time, they could also look at other artworks presented in the exhibition room which were available in the background. As a result, the conditions of reception of art in the gallery might have been considered as more complex than those outside the gallery where artworks were presented one by one. Fourth, there is the possibility that participants read the text a few times, whilst in most cases they listened to the description only once (it occasionally happened that a participant asked that some part of the description he or she did not catch be repeated). Repeated reading of (some parts of) the text may have been particularly likely to occur in the gallery condition (this situational context encourages viewers of the artworks to show an interest [54]) since the best results in the memory test were obtained by participants tested in the gallery who read the contextual information on their own. Admittedly, one could try to control how people get acquainted with the text of the description, e.g. using an oculography, but it would be complicated, especially in gallery conditions. Moreover, such an approach could reduce the ecological validity of the study. Fifth, among the artworks taken into account in this study, there were no remarkably controversial pieces which might cause extremely hostile emotions and aggressive reactions. Critical art also includes works that can be interpreted as e.g. sacrilegious (combining religious and erotic motives) and our research was limited to less controversial artworks. So, our findings should not be generalised on the aesthetic experience of critical art in all its diversity. Sixth, heightened physiological arousal may be partly due to body movement. Therefore, it is possible that the enhanced emotional reactions for the gallery context were due to the fact that people were walking around in the gallery and sitting in the laboratory setting (see e.g. [98]).

## Conclusions

To sum up, for the first time we have shown the enhancing effect of an art gallery on aesthetic experience in the case of critical art (an effect which has previously been described in the context of different visual art movements [51, 54, 55, 57, 66, 67, 69–72, 93]). Although such art might be appraised in a specific way and evoke unpleasant emotions [11, 13], it seems that aversion to this kind of art can be subdued in the situation of receiving artworks in a typical context, i.e. a museum or a gallery. Therefore, in our opinion, it is worthwhile developing research on hostile emotions framed in the theoretical context of the appraisal model for aesthetic emotions [9–12] by conducting further studies in the gallery context. It is possible that viewers are focused on different aspects of controversial artworks which they experience in the exhibition context or in a non-gallery setting (e.g. when viewing the original piece of art in a gallery participants may focus more on the novelty and complexity of the stimulus than when viewing a reproduction of artwork outside a gallery). As a consequence, appraisals, emotions, and action tendencies may differ due to the situational context of perceiving controversial artworks. Regarding the impact of extended contextual information, we found similar effects for liking and subjective understanding of artworks. The interpretative prompts in the form of curatorial descriptions that were made available to the participants contributed both to an increase in understanding and liking of critical art (which was particularly evident when comparing conditions of reading contextual information in the laboratory setting with no curatorial information in the laboratory setting). Therefore, our results are in line with models of art reception which indicate that the meaning-making process is self-rewarding [1, 2, 59–61]. Viewers, especially non-experts in the field of art, need interpretative hints to figure out and appreciate challenging contemporary art [51, 62, 79, 80], including critical art. Learning specialist knowledge of contemporary art, even once the descriptions of the particular artworks are made available (which can be seen as a form of very short training), may result in better understanding of artworks, whereby our study showed that reading a description of an artwork by oneself in a laboratory setting seems to increase understanding more than listening to it. Moreover, this learning process also increases liking of art in non-experts. This effect, which in our study was shown in the laboratory setting when participants read curatorial descriptions on their own, may be explained by taking into account a reward-learning perspective [99]. Knowledge acquisition is a rewarding experience, therefore the acquisition of new knowledge on particular contemporary artworks may not only raise the mood of the audience and cause liking of these artworks. It may also strengthen curiosity and interest in contemporary art in general in the future.

We believe that further research is needed to test the impact of the method of providing contextual information and related cognitive effort on emotions and aesthetic judgements. It would be valuable, for example, to develop a reading procedure so that the participants may not read parts of the text repeatedly (or skip them). Such an approach would overcome a limitation of the current study. Furthermore, it would also be desirable to research the reception of critical art by experts. It seems particularly interesting to test the aesthetic experience of controversial art of experts and non-experts, who additionally differ in their personality traits, such as right-wing authoritarianism (for a similar measurement of individual differences see [11])–to assess whether the reception of critical art (that could be deemed offensive or aversive), depends more on viewers’ specialist knowledge or their values.

## Supporting information

S1 Appendix Curatorial information on works of art taken into account in this study. (DOC)

S2 Appendix Items in the memory test. (DOC)
