# Supplementary material for: The aesthetic experience of critical art: The effects of the context of an art gallery and the way of providing curatorial information
Source: PLoS One. 2021 May 28;16(5):e0250924. doi: 10.1371/journal.pone.0250924 (PMC8162635; doi:10.1371/journal.pone.0250924)
Supplement: S1 Appendix — (DOC) [file pone.0250924.s001.doc]

## S1 Appendix. Curatorial information on works of art taken into account in this study.

Artwork 1. Pamela Bożek, “Lifeline”, 2018, photography, acrylic plates

In June 2018, the Maltese government stated that it had no competence to accept the “Lifeline” lifeboat into the port of Valletta. On board there were 239 people rescued in the Mediterranean. It was not the first or the only situation in which ships which are overcrowded and deprived of humanitarian aid have been sent to another European country. It was all part of the policy of sealing borders. Amongst those rescued there were 14 women. Valletta, like many European capitals, is subject to intense gentrification. As a result, the delightful architecture is directly adjacent to many ruined abandoned tenements. During artistic residence as part of the European Capital of Culture project, the artist’s attention was drawn to the common practice of naming residential buildings – on most tenements on the right side of the front door there is a plate with name of the house or the name of its male or female patron. The artist chose 14 female names popular in Eritrea and Somalia and put them on ordered plates – thus, she named empty homes in Valletta with these names.

*The work is accompanied by “Your Things” fabric created as part of a tailoring workshop of artist’s ideas at the Centre for Foreigners in Łuków. The workshops were carried out thanks to a mini-grant of the FemFund Feminist Fund and in cooperation with “Dla Ziemi” Association. In her artistic practice, the artist often initiates and engages in social activities, which gives her works a participatory character.*

Artwork 2. Marianna Grabska, “I Won’t Pretend I’m not Here”, 2018, ceramics, puzzles, children’s chair.

*“Take responsibility for the face of the world. The symbols of today enable the reality of tomorrow. Notice the swastikas and the other signs of hate. Do not look away, and do not get used to them. Remove them yourself and set an example for others to do so.”
Timothy Snyder, “On Tyranny: Twenty Lessons from the Twentieth Century”, 2017*

Marianna Grabska placed the ARTE ET MARTE (by skill and by a weapon) inscription against the background of a fragment of “Racławice Panorama*” in the form of a puzzle, juxtaposing it with rocking (Pegasus?) horses and a children’s chair. The space of a child’s room and children’s creation is dangerously associated with the “games” of adults – which further emphasises the reference to hussars or an overturned piece of furniture. The artist “plays” with national symbols, asking the question about the role of art in the fight against the dangers of a modern world as promised by the title: “I Won’t Pretend I’m not Here”.

* The oil painting created in 1893-1894 by a group of artists under the supervision of Jan Styka and Wojciech Kossak, depicting the Battle of Racławice (1794), in which the Polish army under the command of General Tadeusz Kościuszko won a victory over the Russian army.

Artwork 3. Agnieszka Mastalerz, ”(H) Reconstruction of Position”, 2018, 1-channel video installation, 8’42”.

The work is a video recording of a few-minutes of choreography inspired by archival photographs from the Memorial Site in Neuengamme in Hamburg. Black and white photos taken in 1945 are 20 topographic half-close-ups of the chests of 9 girls and 11 boys – children of Bullenhuser Damm. Each of them has their right or left arm raised; sometimes you can see the hand of an adult man who influences the position of the photographed child. In two looped shots of a few minutes – close-ups and general plans – the 11-year-old girl repeats after an invisible person a similar sequence of positions involving an upper part of her body. The background of the choreography is the interior of the former Bersohn and Bauman Children’s Hospital at Sienna Street in Warsaw in the 1940s, which remained in the small ghetto (the building was chosen as the main base for the future Warsaw Ghetto Museum). The letter “H” in the title refers to the only child whose full name is unknown.

Artwork 4. Gustaw Maj, “Radical Urban Toys”, 2018/2019, objects.

“Radical Urban Toys” are mechanical sculptural objects created as a result of the creative use of upcycling. These objects are a practical tool with wide application for urban guerrillas and in urban activism; they are also used for the symbolic recovery of urban space. They are a tool of creative discord arising from the need to confront current reality. They can be used to intervene in urban space, to stimulate an audience’s ability to self-reflect and see critically. They are also used to jam the corporate message that it is an interference in the sphere of decision-making of individuals. Thanks to telescopic poles, on which some pieces were built, the objects are an extension of the user’s body and allow intervention in spaces that are difficult to access. The function of some of them is properly camouflaged so as not to reveal their destiny to bystanders. They are directed against ubiquitous control – one of the objects is used to neutralise surveillance cameras. Although for some people empty spray cans can be associated with urban vandalism, recent events in Hong Kong are the best proof of how similar strategies can be useful for practical implementation of the idea of civil disobedience.

Artwork 5. Agnieszka Sejud, “HOAX”, 2019, photography, collage.

*“Hoax” is a photographic project showing a subjective vision of the present in Poland. Political events after the 2015 elections played a significant role in its creation – they aroused rebellion and discord, giving a critical dimension to the work. The artist writes that working on the project helped her to get rid of shame because of her origin and sense of inferiority to the West. Poland is still different from Europe. Demands for the separation of the state from the Catholic Church are considered radical and called anti-clericalism. The artist draws attention to the privileged position of the Church, the hypocrisy of the faithful, inequality between men and women, homophobia, xenophobia, and the upcoming ecological disaster. The present is a universe of populism, post-truth, and fake news. Politics has strongly invaded the lives of Poles in recent years, raising attitudes to citizenship. According to Sejud, it is difficult to resist the impression that Poland looks like HOAX, which in English means mystification, fraud, joke, and as a borrowing in Polish it acts as false information that simulates true information in order to mislead the recipient.*

Artwork 6. Daniel Kotowski, Untitled, 2018, installation, video, 1’52’’.

Daniel Kotowski’s work refers to the concept of bio power (power over biology) and biopolitics (conscious use of bio power in practice) in the view of Michel Foucault. The artist was inspired by the apparent contradiction he noticed in the fact that the matter of glass comes from the same source as that of stone. Glass is made of quartz sand, which is made of naturally crushed quartz stone. The object indicates the relationship between stone and glass: the first element expresses durability, opacity and roughness, the second – delicacy, transparency and smoothness. Glass is the result of human production, which can symbolise utopia and bio-power, while stone belongs to the sphere of nature and is not consistent with the theory of bio-power. The artist raises the proposition of Michel Foucault not calling the stone “sick” – hence the utopian attempt to perfectly combine both materials, despite their visual contrast. Despite visual contrast, the artist makes a utopian attempt to perfectly combine both materials to maintain a balance between both spheres.

Artwork 7. Dominika Piętak, “BIŁ-GO-RAJ (BEAT-HIM-PARADISE)”, 2019,
installation.

The work consists of two boards of the city plan of Biłgoraj, which is divided with a band of moss. The boards were found in a former barn in the artist’s yard. Her family reports show that during the Polish People’s Republic (PRL) a two-part plan decorated Biłgoraj market. The key word of this work is paradise. In 1606, a song “Poland is a paradise for Jews, hell for peasants, heaven for the nobility” was published in Latin, with grossly anti-Semitic overtones. The vision of Poland as Paradisus Judaeorum is a myth that authorities are happy to also use nowadays. The work refers to this myth, which refutes the evidence of forest murders of Jews by Poles.

Artwork 8. Aleksandra Liput, “DIY Totems”, 2019, installation.

According to epigenetics, we inherit trauma and anxiety through cellular memory called metabolic or epigenetic, which affects our health and perception and sensation of reality. The people examined noted a different way of secreting the stress hormone, which causes anxiety disorders – these changes are visible until the third generation. An adequate distance from events allows you to deal with trauma and stop passing it on. Instead of traditional ways of dealing with hereditary traumas (e.g. researching family roots or psychotherapy), the artist focuses on forgotten and displaced ways or ways rejected by rationalists and Catholics. She recalls shamanic theories, magic rituals, and faith in the healing power of stones and animals. The installation includes: “Shaman Totem” with a rock crystal with a strong cleansing effect in its main point, “Snake Totem” – one of the oldest healing symbols, dream catcher – an amulet that is a part of the beliefs of North American Indians, and hands placed on the floor that symbolise healing through touch and the magic of contact.

Artwork 9. Sylwia Brzyszczyk, “Reading from the Letter to Subjects”, 2019, video, 4’27’’.

The film raises the problem of stereotypes related to the role of women and men in the modern world, their thoughtless duplication and indoctrination with regard to children from an early age with the help of toys adapted to their gender. The artist by contrast juxtaposes the image of a girl playing with a doll with controversial views about women and their place in society – the innocence of a carefree child emphasises the absurdity and the terrifying tone of the quoted words. Although, the excerpts seem to come from the distant past, apart from fragments from the letters of St. Paul, they are taken from texts created in the 21st century, i.e. from the “Wędrując ku dorosłości” (“Wandering Towards Adulthood”) textbook on bringing up families for students of grades 1-3 of junior high school by Teresa Król and from fragments of statements by Paweł Murziński, a priest of the Białystok diocese and PhD of moral theology. The juxtaposition of these statements makes them ridiculous gibberish. In combination with the image that is presented, it draws attention to the fact that children learn by observing and repeating the behaviour of adults, reproducing models adopted in everyday life.

Artwork 10. Magdalena Hoffa, “2 399 073”, 2016, installation.

The title of the installation refers to the total number of people killed by God in the Old Testament, the holy book of Judaism and Christianity – the followers of these religions make up nearly 2.5 billion of the 7.3 billion human population (data from 2016). It means that around 1/3 of people living on Earth receive spiritual support from a book full of descriptions of violent events. The artist was also inspired by current world events (including the migration crisis) and their impact on changing European and Polish mentality – including fascinating ethical attitudes in which religious education plays a huge role. The installation asks various questions on many important matters: the issue of interpretation of the Bible – as both a cultural and holy text, the role of the Church in transmitting the word of God, the issue of manipulating another person using religious texts for political or ideological purposes. It is written in the Bible that God created man in his own image and likeness. Or maybe, as Ludwig Feuerbach, a 19th-century German philosopher, wrote it is exactly the opposite and it is man who creates God by creating its mirror image?

Artwork 11. Justyna Lach, “Land Canoe”, 2019, installation.

Reflections on madness, which are always carried out from a distant, alien position of reason, form the starting point for this work. Madness is understood here as a disease, behaviour or way of thinking that goes beyond normality and so-called common sense. Justyna Lach refers to the problem as seen by Michel Foucault in “A History of Insanity in the Age of Reason”, and symbolically to the history of functioning of the so-called ships of fools in medieval Europe that served to exclude people who did not meet certain social norms. Going beyond the area and language of modern psychiatry, Lach makes a philosophical reflection on the sense and meaning of divisions of personality of an individual or society into oppositional areas. “Land Canoe” makes it possible to navigate the mental landscape, where lands are usually identified with the environment of reason and waters with the territory belonging to madness. The artist sets a canoe as a symbol of a senseless dispute about dominance and a lack of mutual understanding of oppositional attitudes and views.
