# Supplementary material for: The aesthetic experience of critical art: The effects of the context of an art gallery and the way of providing curatorial information
Source: PLoS One. 2021 May 28;16(5):e0250924. doi: 10.1371/journal.pone.0250924 (PMC8162635; doi:10.1371/journal.pone.0250924)
Supplement: S2 Appendix — (DOC) [file pone.0250924.s002.doc]

**S2 Appendix. Items in the memory test.**

Note: Correct responses are marked as *.

1. What two materials are combined in Daniel Kotowski's artwork *Untitled*?

A. stone and quartz

B. quartz and opal

C. quartz and talc

D. stone and glass*

2. The work *Land Canoe* by Justyna Lach makes you think about

A. the limitations that are placed on human beings

B. dividing society into opposing groups*

C. the resources that each human being possesses

D. both answers A and C are correct

3. The installation *DIY Totems* by Aleksandra Liput includes

A. totems with healing symbols*

B. objects used for suicide

C. items associated with male and female gametes

D. all answers are correct

4. What does the inscription ARTE ET MARTE used by Marianna Grabska in her work entitled *I Won’t Pretend I’m not Here* mean?

A. art for art's sake

B. skill and weapon*

C. art is the supreme value

D. none of the answers is correct

5. In the photographic project *HOAX* by Agnieszka Sejud, the artist presents

A. a subjective vision of the present in Poland*

B. Polish culture and tradition

C. the reality of Poland in the 1990s

D. both answers A and B are correct

6. The letter 'H' in the title of the work *(H) Reconstruction of Position* by Agnieszka Mastalerz refers to

A. the first letter of a Jewish child's name*

B. the first letter of a ballet routine

C. the first letter of the word 'hospital'

D. both answers A and C are correct

7. The installation *BIŁ-GO-RAJ (BEAT-HIM-PARADISE)* by Dominika Piętak presents

A. picturesque areas of the town of Biłgoraj

B. a map of the town of Biłgoraj*

C. locations of monuments in the town of Biłgoraj

D. all answers are correct

8. In the work *Radical Urban Toys* by Gustaw Maj, telescopic sticks and spray paints are tools used for

A. urban vandalism

B. learning to create street art

C. fighting the corporate system*

D. none of answers are correct

9. Aleksandra Liput's installation *DIY Totems* refers to problems related to

A. infertility

B. trauma and anxiety*

C. lack of acceptance

D. all answers are correct

10. The strand of moss running along Dominika Piętak's installation *BIŁ-GO-RAJ (BEAT-HIM-PARADISE)* symbolises

A. the forest murders of Jews carried out by Poles*

B. plant species in danger of extinction

C. areas where forest has been removed

D. both answers B and C are correct

11. In the work *Land Canoe* the artist Justyna Lach focuses on

A. overcoming barriers and satisfying curiosity about the world

B. conflict for domination and lack of mutual understanding of opposing views*

C. journeys into the depths of human nature

D. both answers A and C are correct

12. The plaques placed by residential buildings in the work *Lifeline* of Pamela Bożek describe

A. the birth and wealth of the householder

B. the happy life of their inhabitants

C. the names of the houses or their patron saints*

D. none of the answers is correct

13. What problem is raised by Sylwia Brzyszczyk in her video *Reading from the Letter to Subjects* showing a girl playing with a doll?

A. children's consumerist approaches to the world

B. stereotypes connected with children

C. disapproval of other people, which can be seen in young children

D. stereotypes connected with the role of men and women nowadays*

14. The work *Radical Urban Toys* by Gustav Maj is meant to provoke reflection in the viewer about

A. increasing control over individuals*

B. growing street vandalism

C. enriching the appearance of cities through street art

D. none of the answers is correct

15. According to Sylwia Brzyszczyk, the author of the artwork *Reading from the Letter to Subjects*, children learn by

A. observing and repeating adult behaviour*

B. the influence of the mass media

C. activity and free play

D. both answers A and B are correct

16. The choreography in the work *(H) Reconstruction of Position* by Agnieszka Mastalerz depicts

A. an attempt to reproduce the movements of Jewish children*

B. a difficult ballet dance lesson in a Russian school

C. performing punishment in front of a torturer

D. none of the answers is correct

17. What was Magdalena Hoffa's inspiration for the work entitled *2 399 073*?

A. the impact of the migration crisis and other current world events on the change in European and Polish mentality*

B. deep conviction about the injustice of life

C. the desire to show people that the judgements of God are impenetrable

D. all answers are correct

18. What was the aim of Daniel Kotowski's work *Untitled*?

A. to show the perfect harmony between the two materials used

B. an attempt to combine the two materials used despite their contrasts*

C. to show the striking mismatch between the two materials used

D. none of answer is correct

19. The title of the work *HOAX* by Agnieszka Sejud refers to

A. rich cultural possessions

B. false information that misleads the viewer*

C. the influence of religion on Polish culture

D. none of the answers is correct

20. What does the number in the title of Magdalena Hoffa's installation *2 399 073* refer to?

A. the total number of people who die in the New Testament

B. the total number of people called to follow Christ in the Bible

C. the total number of people who die by the hand of God in the Old Testament*

D. the total number of refugees who died in the previous year
